# Sirtinol Inhibits Neutrophil Elastase Activity and Attenuates Lipopolysaccharide-Mediated Acute Lung Injury in Mice

**DOI:** 10.1038/srep08347

**Published:** 2015-02-10

**Authors:** Yung-Fong Tsai, Huang-Ping Yu, Wen-Yi Chang, Fu-Chao Liu, Zhen-Cheng Huang, Tsong-Long Hwang

**Affiliations:** 1 Graduate Institute of Natural Products, School of Traditional Chinese Medicine, College of Medicine, Chang Gung University, Kweishan 333, Taoyuan, Taiwan; 2 Graduate Institute of Clinical Medical Sciences, College of Medicine, Chang Gung University, Kweishan 333, Taoyuan, Taiwan; 3 Department of Anesthesiology, Chang Gung Memorial Hospital, Taoyuan, Taiwan; 4 Chinese Herbal Medicine Research Team, Healthy Aging Research Center, Chang Gung University, Kweishan 333, Taoyuan, Taiwan

## Abstract

Enhanced activity of neutrophil elastase leads to a protease–antiprotease imbalance, and plays an essential pathogenic role in acute lung injury (ALI) and acute respiratory distress syndrome. We assayed the pharmacological effects and mechanisms of the action of sirtinol in human neutrophils, and in neutrophil elastase (HNE)-induced paw edema and lipopolysaccharide (LPS)-mediated ALI in mice. Sirtinol significantly inhibited the activity of HNE from human neutrophils in response to various stimulators. The inhibitory effects on HNE activity were not mediated through protein kinase A, calcium, extracellular-regulated kinase, c-Jun N-terminal kinase, p38 mitogen-activated protein kinase, Akt, or Src family kinases. Analysis of enzymatic activities showed that sirtinol inhibited HNE activity in a concentration-dependent manner. These results demonstrate that sirtinol does not affect neutrophil function and is an HNE inhibitor. In addition, administration of sirtinol significantly inhibited HNE-induced paw edema, and attenuated the myeloperoxidase activity and reduced pulmonary wet/dry weight ratio in the LPS-induced ALI mouse model. Our study indicates that sirtinol has anti-inflammatory effects through direct inhibition of HNE activity and attenuates HNE-induced and LPS-mediated tissue or organ injury in vivo. Sirtinol is a novel HNE inhibitor and may have the potential for clinical application in the treatment of inflammatory lung diseases.

Acute lung injury (ALI) and its most severe form, acute respiratory distress syndrome (ARDS), have high morbidity and mortality rates, with an overall mortality estimated at 46%^1^. These life-threatening diseases can result from bacterial infection, trauma/hemorrhagic shock, chemical inhalation, blood transfusion, and ventilator-associated or aspiration pneumonia. They are characterized by large infiltrates of activated neutrophils, disruption of the alveolar capillary barrier, high levels of release of reactive oxygen species and proteases, and an impaired gas exchange in the respiratory tract^2^. Numerous studies have reported that neutrophils and neutrophil elastase (NE) are the main inflammatory mediators implicated in acute alveolar injury and interstitial edema related to an NE-mediated increase in vascular permeability^3^.

NE plays a critical role in the initiation and progression of pulmonary inflammation. It contributes to neutrophil migration toward the inflammatory site, where the recruited activated neutrophils degranulate, releasing more elastolytic proteases to degrade the proteins of invading pathogens or injure the elastin-rich connective tissue of lungs^4^,^5^. Convincing evidence has confirmed that excessive NE is an essential contributor to the progression of ALI or ARDS, and its deleterious actions are obvious. 1-Antitrypsin is an endogenous secretory elastase inhibitor that is abundant in the peripheral alveolae. It naturally protects lung tissues from elastolysis, and tightly maintains a balanced elastase–antielastase relationship^6^. Besides its role in ALI and ARDS, impaired antielastase protection also initiates the development of numerous chronic lung diseases, including chronic obstructive pulmonary disease, asthma, and cystic fibrosis^7^,^8^. Although 1-antitrypsin has been successfully used to treat premature emphysema caused by 1-antitrypsin deficiency^9^, the supply of purified human 1-antitrypsin is usually insufficient to meet patient demand. There are few NE inhibitors that are approved for clinical use. Sivelestat sodium hydrate (ONO-5046) was synthesized to be a selective human neutrophil elastase (HNE) inhibitor, and is indicated to treat ALI resulting from pathogen infections^10^. However, clinical trials of its therapeutic efficacy in human ALI and ARDS have produced discordant results^11^. In addition, sivelestat is not a popular choice because of its risks of organ toxicity and poor pharmacokinetics, so the design or invention of a new HNE inhibitor for clinical use is required.

Sirtinol (2-[(2-hydroxynaphthalen-1-ylmethylene)amino]-N-(1-phenethyl) benzamide) (Calbiochem, La Jolla, CA, USA) is known to be an inhibitor of sirtuin 2, a nicotinamide adenine dinucleotide-dependent deacetylase. Sirtuin activity is involved in gene expression, metabolic regulation, cell apoptosis, and aging, and sirtuin 2 inhibitors are thought to be useful in the treatment of cancer and neurodegenerative diseases^12^. Recently, we discovered an unexpected anti-inflammatory effect of sirtinol. We showed that, in rats, sirtinol treatment greatly reduced neutrophil infiltration and cytokine production in lung and liver after trauma/hemorrhage injury^13^,^14^. However, it remains unknown whether sirtinol directly affects neutrophil functions, especially the activity of released NE, and whether administration of sirtinol can protect mice from HNE- and lipopolysaccharide (LPS)-induced tissue damage. We hypothesized that sirtinol inhibits HNE activity to attenuate LPS-induced lung injury and HNE-induced paw edema.

In this study, we assayed the inhibitory activity of sirtinol in vitro and in vivo. We established a cellular model of isolated human neutrophils to evaluate the anti-inflammatory functions of sirtinol. Significantly, our results suggest that sirtinol inhibits the activity of HNE, but does not alter cell function. Moreover, administration of sirtinol reduces HNE-induced paw edema and attenuates LPS-induced ALI in mouse models.

## Results

### Sirtinol significantly inhibits HNE activity in N-formyl-methionyl-leucyl-phenylalanine (fMLF)-activated human neutrophils, but fails to inhibit superoxide generation

**Figure 1:**
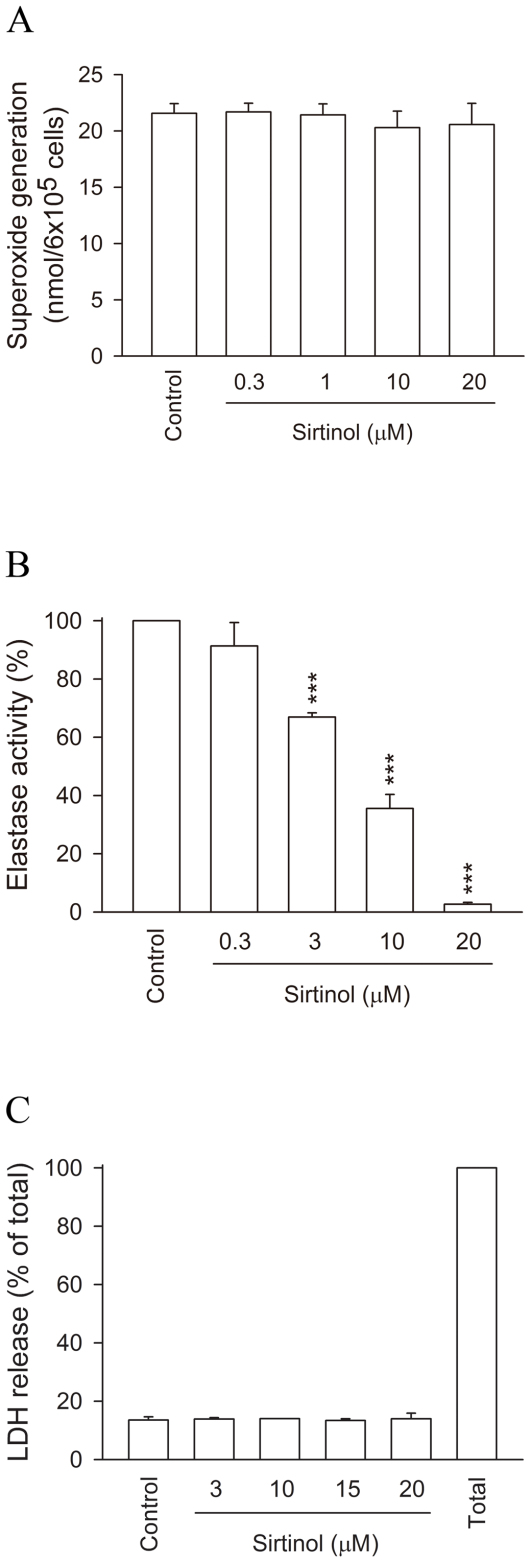
Effects of sirtinol on superoxide release and HNE activity in fMLF-activated human neutrophils. Human neutrophils (6 × 10^5^ cells/mL) were incubated with DMSO (as control) or sirtinol (0.3–20 μM) for 5 min before being activated with fMLF/CB. (A) Superoxide production by SOD-inhibitable cytochrome *c* was measured using a spectrophotometer at 550 nm. (B) HNE activity was measured using methoxysuccinyl-Ala-Ala-Pro-Val-p-nitroanilide as the substrate by continuous analysis of absorbance at 405 nm for 10 min. (C) Human neutrophils were incubated with DMSO (as control) or sirtinol (0.3–20 μM) for 15 min. LDH release into cell-free medium was measured using an ELISA kit, as described in Materials and Methods. Cytotoxicity was evaluated by LDH release as a percentage of total LDH release. All data are expressed as the mean ± S.E.M. (n = 3–5). *** *p* < 0.001 compared with the control.

Superoxide generation and HNE release are considered to represent the major neutrophil functions of respiratory burst and degranulation that occur in activated human neutrophils. When fMLF-stimulated neutrophils were incubated with sirtinol, it had no effect on superoxide generation (Fig. 1A). However, sirtinol (0.3–20 μM) exerted a significant concentration-dependent inhibition of HNE activity in fMLF-induced human neutrophils, with an IC50 value of 6.05 ± 0.65 μM (Fig. 1B).

Sirtinol (3–20 μM) did not cause any cytotoxicity as measured by LDH release assay (Fig. 1C). This cytotoxicity study ruled out the possibility that the inhibition of HNE activity by sirtinol was caused by cytotoxic effects. We also evaluated the antioxidant effect of sirtinol in a cell-free system using the DPPH radical-scavenging assay. Sirtinol (0.3–20 μM) showed no scavenging effect on free radicals, while an effect was seen with the positive control α-tocopherol (vitamin E) (data not shown).

### Protein kinase A (PKA) and protein phosphatase (PP) do not mediate the inhibitory effect of sirtinol

**Figure 2:**
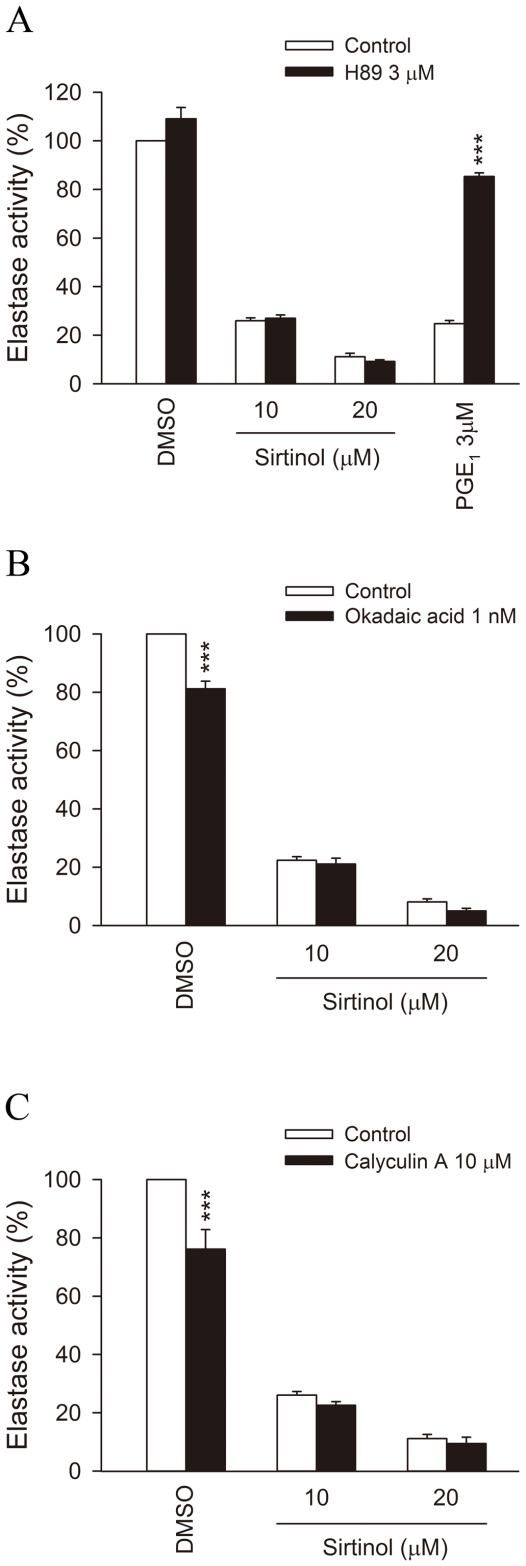
Effects of inhibitors of protein kinase A (PKA) and protein phosphatase (PP) on sirtinol-induced inhibition of HNE activity. Human neutrophils were preincubated for 5 min with (A) H89 (3 μM), (B) okadaic acid (1 nM), or (C) calyculin A (10 nM) before the addition of sirtinol (0.3–20 μM) or PGE_1_ (3 μM). Cells were induced by fMLF/CB for 10 min, and HNE activity was measured spectrophotometrically at 405 nm. All data are expressed as the mean ± S.E.M. *** *p* < 0.001 compared with the corresponding control.

The following experiments were designed to determine which pathways were involved in the inhibitory effects of sirtinol. Previous studies have shown that cAMP/PKA has significant negative regulatory effects on human neutrophil activation and can inhibit HNE release^15^,^16^,^17^. To elucidate whether cAMP/PKA mediates the inhibitory effect of sirtinol, a PKA inhibitor, H89, was used. H89 (3 μM) was able to reverse the inhibitory effect of prostaglandin E_1_ (PGE_1_) but not that of sirtinol (10 μM or 20 μM) (Fig. 2A). These results suggest that PKA does not mediate the inhibitory effect of sirtinol.

Regulation of the phosphorylation state of proteins by PP affects the functions of activated neutrophils and mast cells^18^,^19^,^20^. Both calyculin A (10 nM, a PP1 and PP2A inhibitor) and okadaic acid (1 nM, a PP2A-selective inhibitor) failed to reverse the sirtinol-mediated inhibitory effect on fMLF-induced HNE activity (Fig. 2B and 2C), suggesting that the inhibitory effect of sirtinol is not mediated through regulation of PP1 and PP2A.

### Sirtinol fails to decrease fMLF-activated Ca^2+^ mobilization in human neutrophils

**Figure 3:**
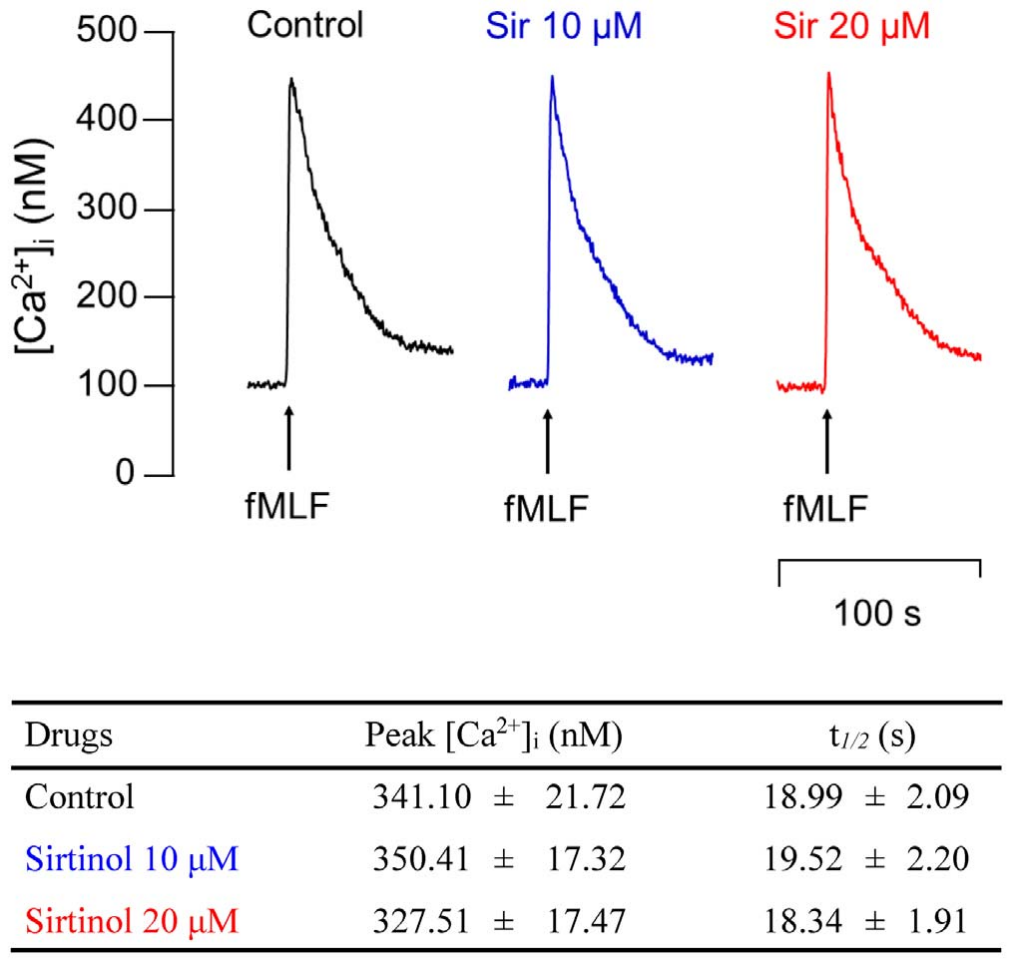
Effects of sirtinol on Ca^2+^ mobilization in fMLF-activated neutrophils. Fluo-3/AM-labeled human neutrophils were treated with DMSO (as control), and sirtinol (10 μM or 20 μM) for 5 min; then cells were activated by fMLF (0.03 μM) and mobilization of Ca^2+^ was determined in real time in a spectrofluorometer. Representative traces are shown. Peak intracellular calcium concentration ([Ca^2+^]_i_) and reduction of the time taken to decline to half of its peak values (t*1/2*) are shown as mean ± S.E.M.

When human neutrophils are activated by chemoattractants, the concentration of intracellular Ca^2+^ increases transiently. The transient increase of [Ca^2+^]_i_ leads to activation of neutrophils, and depression of the magnitude or duration of the increase is able to inhibit superoxide generation or NE release^21^,^22^,^23^. Here, administration of sirtinol (10 μM or 20 μM) did not affect the peak concentration of [Ca^2+^]_i_ and the time taken to decline to half of its peak values (t*_1/2_*) in fMLF-activated human neutrophils (Fig. 3).

### Sirtinol does not attenuate phosphorylation of Akt, MAPKs, and Src family kinases in fMLF-activated neutrophils

**Figure 4:**
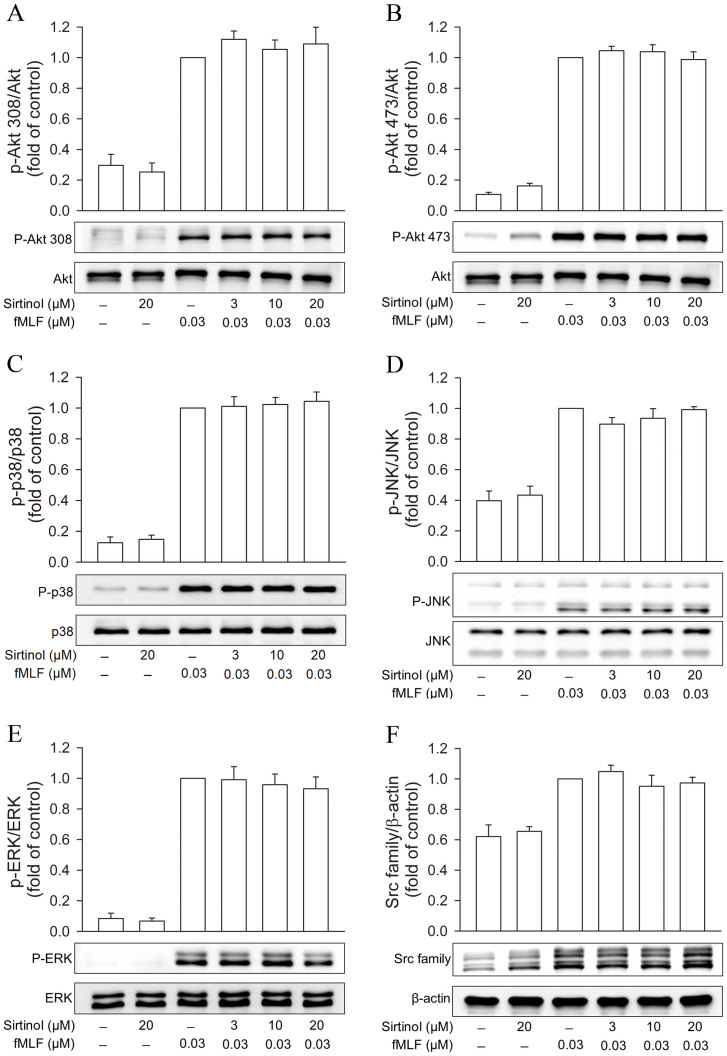
Effects of sirtinol on phosphorylation of Akt, MAPKs, and Src family kinases in fMLF-activated human neutrophils. Human neutrophils were incubated with sirtinol (3, 10, or 20 μM) for 5 min before stimulation with or without fMLF (0.03 μM) for another 0.5 min. All the Western blotting experiments were performed under the same condition. After transferring the blots onto nitrocellulose membranes, we immediately cropped the targeted blots according to referenced indicating markers, and then targeted proteins were immunoblotted with its specific monoclonal antibody. Results for Western blots using anti-phospho antibodies directed against (A) Akt308, (B) Akt473, (C) p38 MAPK, (D) JNK, (E) ERK, and (F) Src family kinases are shown. Bands on the blots were analyzed using a densitometer, and the quantitative ratios for all samples were normalized to the corresponding total protein or to β-actin. All data are expressed as mean ± S.E.M. (n = 4–5).

To investigate whether other fMLF-mediated downstream signals were affected by sirtinol, activation of Akt, MAPKs, or Src family kinases was analyzed by Western blot using an antibody specific for each of their corresponding phosphorylated forms. Stimulation of human neutrophils with fMLF caused rapid phosphorylation of Akt, p38 MAPK, JNK, ERK, and Src family kinases. However, sirtinol (3, 10, or 20 μM) showed no inhibitory effect on the phosphorylation of these proteins (Fig. 4).

### Sirtinol specifically inhibits HNE activity, but not superoxide generation, in response to different stimulants

**Figure 5:**
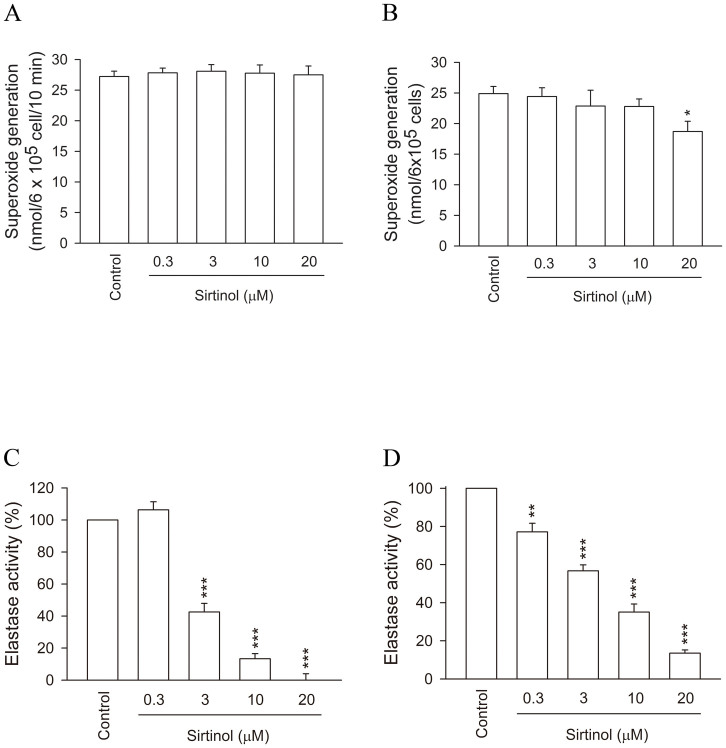
Effects of sirtinol on superoxide production and HNE activity in human neutrophils activated by stimulants other than fMLF. Human neutrophils (6 × 10^5^ cells/mL) were incubated with DMSO (as control) or sirtinol (0.3–20 μM) for 5 min before being activated. Cells were activated with (A) PMA and (B) MMK1/CB, and superoxide release was measured. Cells were activated with (C) MMK1/CB and (D) PAF/CB, and HNE activity was measured. All data are expressed as mean ± S.E.M. (n = 3–7). ** *p* < 0.01; *** *p* < 0.001 compared with the control.

We also tested the effect of sirtinol on the major neutrophil functions in cells treated with other stimulants. When neutrophils were incubated with sirtinol, the sirtinol had no effect on superoxide generation in phorbol myristate acetate (PMA)-stimulated neutrophils (Fig. 5A); and only a light inhibition at higher concentrations in Leu-Glu-Ser-Ile-Phe-Arg-Ser-Leu-Leu-Phe-Arg-Val-Met (MMK1)-activated cells (Fig. 5B). However, sirtinol (0.3–20 μM) exerted a significant concentration-dependent inhibition of HNE activity in MMK1- and PAF-treated human neutrophils, with IC_50_ values of 2.45 ± 0.37 and 4.47 ± 0.76 μM, respectively (Fig. 5C and 5D).

### Sirtinol directly influences the enzymatic activity of HNE

**Figure 6:**
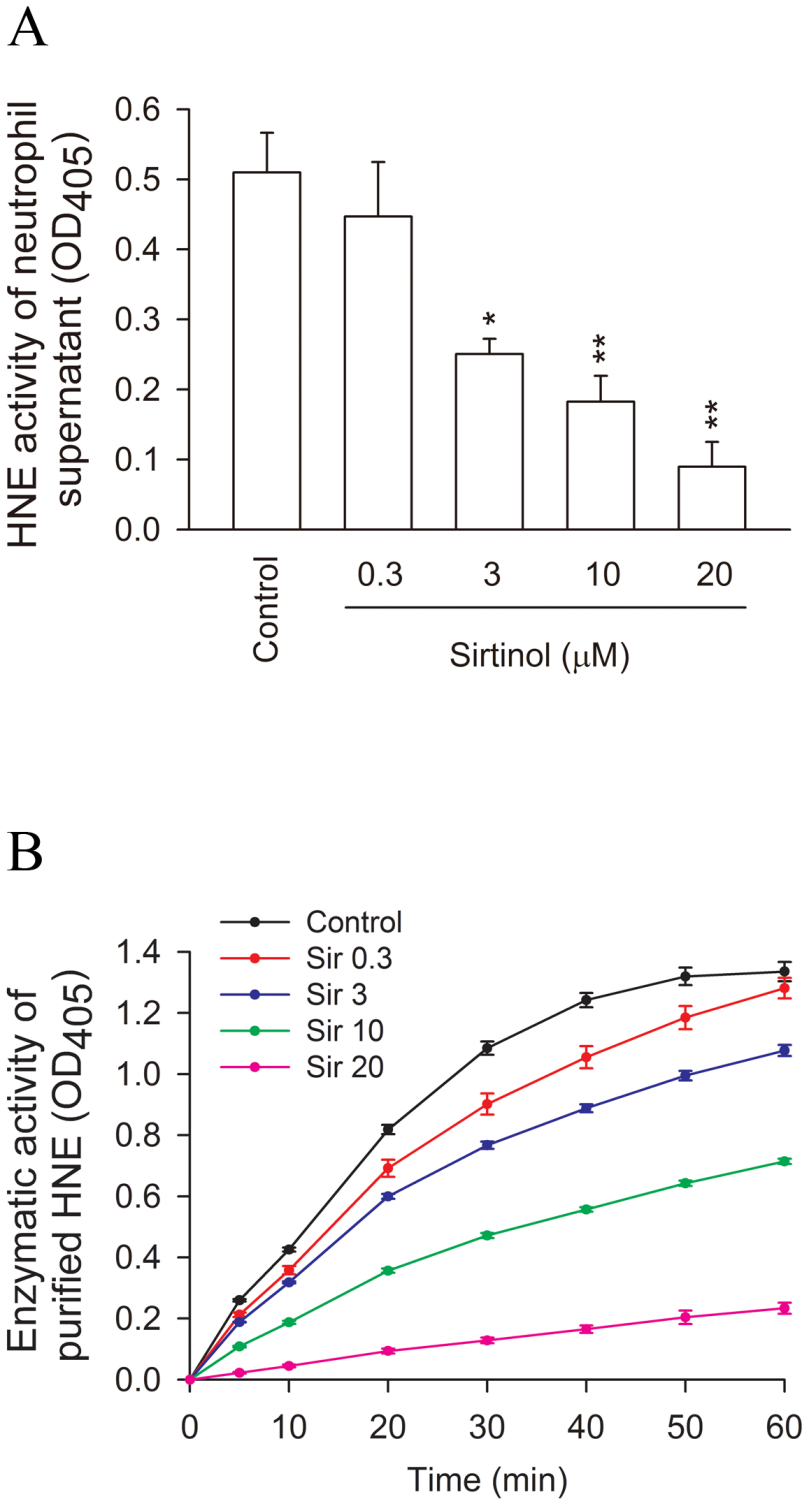
Effects of sirtinol on the activities of HNE in cell-free systems. (A) Conditioned medium was obtained from the supernatant of human neutrophils that had been activated by fMLF/CB. The inhibitory effects of sirtinol on the activity of HNE were detected by spectrophotometry at 405 nm. (B) Purified HNE was used to assay enzymatic activity. All data are expressed as the mean ± S.E.M. (n = 3–4). * *p* < 0.05, ** *p* < 0.01 compared with the control.

The effects of sirtinol were further evaluated by adapting an HNE in vitro activity assay to determine whether sirtinol directly inhibits HNE activity. First, we tested the inhibitory effect of sirtinol using conditioned medium derived from the supernatant of activated human neutrophils. Sirtinol clearly reduced the activity of HNE with an IC_50_ value of 6.37 ± 1.37 μM (Fig. 6A). We validated this inhibition by sirtinol using purified HNE. Consistent with the previous result, sirtinol showed significant inhibition of the enzymatic activity of HNE in a concentration-dependent manner, with an IC_50_ value of 8.40 ± 0.53 μM (Fig. 6B). These data confirmed that sirtinol directly inhibits HNE activity.

### Sirtinol inhibits HNE-mediated paw edema in C57BL/6 mice

**Figure 7:**
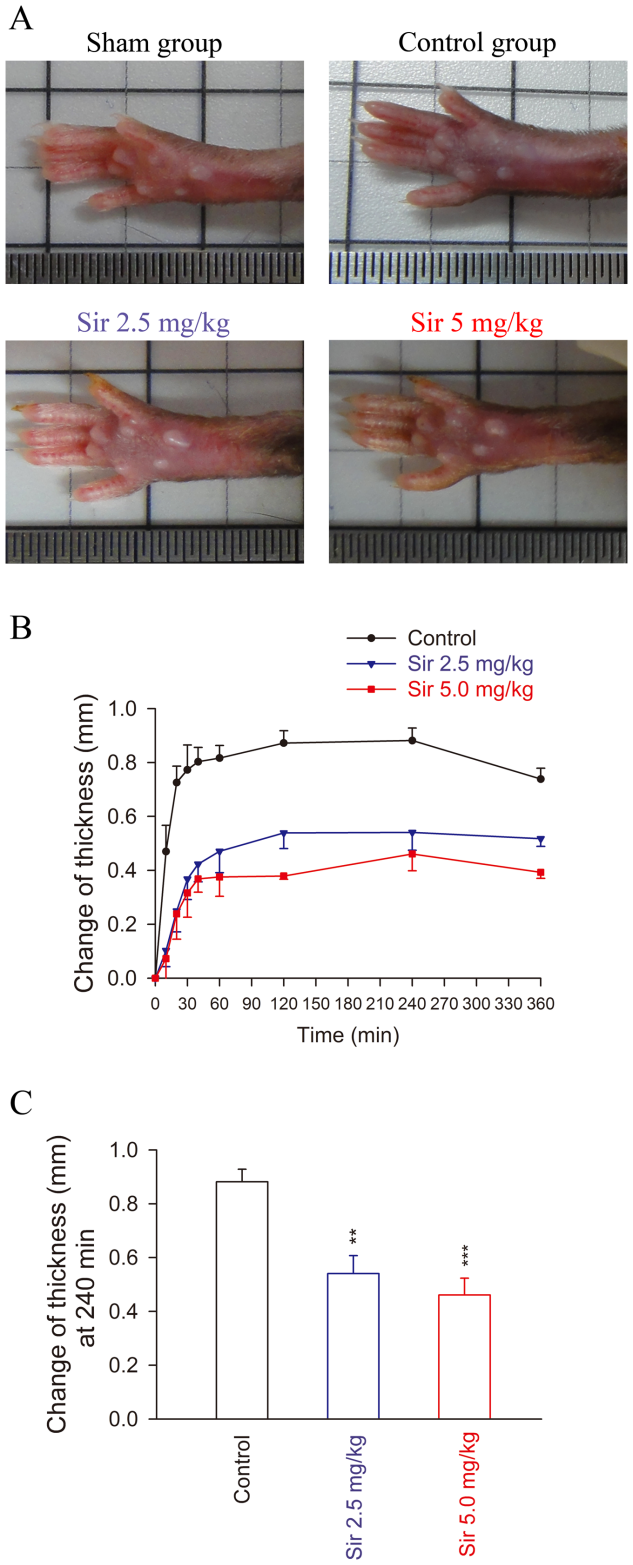
Effects of sirtinol on HNE-induced paw edema in C57BL/6 mice. Animals were injected with sirtinol (2.5 mg/kg or 5 mg/kg body weight, intraperitoneally) or an equal volume of the vehicle (Veh; 50 μL, 10% DMSO). One hour later, paw inflammation was induced by injecting 25 μL of 200 μg/mL HNE or 0.9% saline (sham-treated group) into the intraplantar space in the right hind paw. (A) Representative photos of paw edema are shown. (B) The thickness of the paw was measured with a digital Vernier caliper at the indicated times after HNE injection. (C) The thickness of paw edema at 240 min after induction was shown. All data are expressed as mean ± S.E.M. ** *p* < 0.01; *** *p* < 0.001 compared with the control.

Edema was calculated as the average difference of paw thickness (mm) in treated groups compared with that in sham-treated groups (normal saline-induced edema) and the corresponding baseline reference. The intraplantar injection of 25 μL of HNE caused an increase in the thickness of mouse paws. The paw edema peaked at 240 min after induction, and the thickness was 0.88 ± 0.05 mm in the control group. In the sirtinol treatment groups (Sir, 2.5 or 5.0 mg/kg body weight), the mean peak thicknesses were 0.54 ± 0.07 mm and 0.46 ± 0.06 mm, respectively, both of which indicated significantly attenuated paw edema compared with the control group (*p* < 0.01) (Fig. 7).

### Sirtinol attenuates LPS-induced ALI in mice

**Figure 8:**
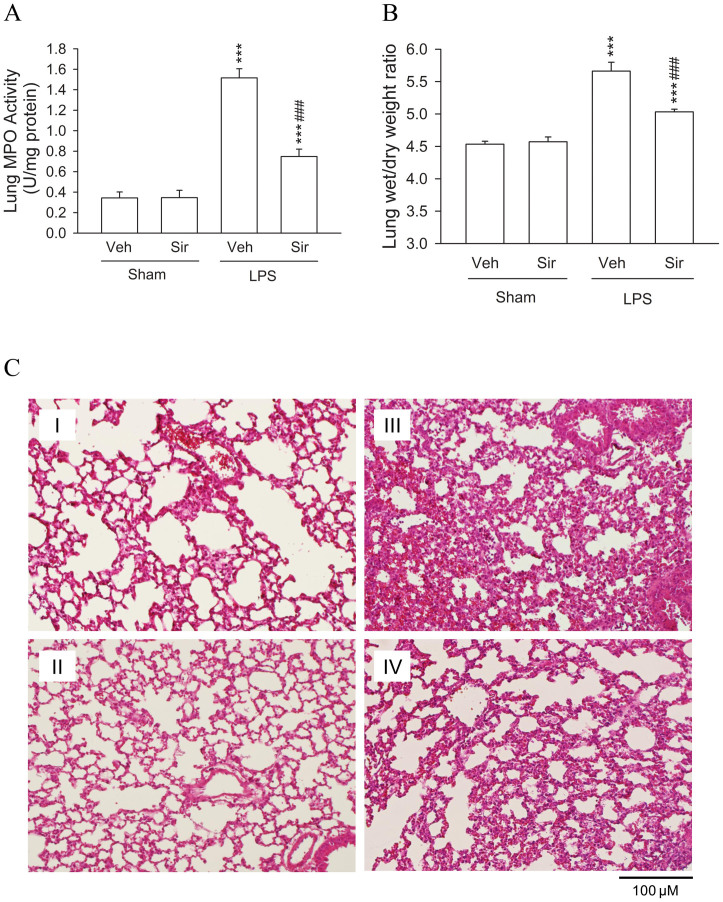
Effects of sirtinol on MPO activities, wet- to dry-weight ratios, and histopathologic features of lungs in mice after a sham operation or LPS-induced acute lung injury (ALI). C57BL/6 mice were treated with sirtinol (5 mg/kg body weight, intraperitoneally) or an equal volume of the vehicle (Veh; 50 μL 10% DMSO). At 6 h after instilling LPS or 0.9% saline into the trachea, mouse lungs were harvested for measurement of (A) MPO activities and (B) wet- to dry-weight ratios, as described in Materials and Methods. All data are expressed as the mean ± S.E.M. for six mice in each group. *** *p* < 0.001 compared with the sham group; ### *p* < 0.001 compared with the ALI + Veh group. (C) Representative photomicrographs of lungs from (I) sham-operated mice injected with vehicle, (II) sham-operated mice injected with sirtinol, (III) ALI animals injected with vehicle, and (IV) ALI animals injected with sirtinol. Sirtinol significantly attenuated pulmonary damage assessed at 6 h after LPS-induced ALI. The representative lung sections are shown stained with hematoxylin–eosin, and at a magnification of ×200.

LPS induced a significant increase in MPO activity, a marker of neutrophil infiltration, in the lungs compared with that in sham-operated animals (Fig. 8A). Treating animals with sirtinol (5 mg/kg body weight, intraperitoneally) clearly attenuated the increase in pulmonary MPO activity after LPS-induced ALI (Fig. 8A). We evaluated the severity of pulmonary edema by calculating the wet- to dry-weight ratios of mouse lungs before and after ALI. The wet- to dry-weight ratios of lung samples from ALI mice were higher than those from sham-operated groups (Fig. 8B). Sirtinol greatly decreased the increase in wet- to dry-weight ratios of lungs that was caused by LPS-induced ALI (Fig. 8B). The histopathologic features of lungs from the four groups were evaluated by light microscopy, and results for sham-operated mice receiving vehicle (Fig. 8C, I) or sirtinol (Fig. 8C, II), and LPS-induced ALI mice treated with DMSO (Fig. 8C, III) or sirtinol (Fig. 8C, IV) are shown. These data show that sirtinol reduced LPS-induced injury in the lungs, although the damage remained greater than in sham-operated animals.

## Discussion

Activated neutrophils play a critical role in tissue damage. Lung dysfunction caused by overactive inflammatory responses is mostly a consequence of excessive neutrophil infiltration and activation. There is considerable evidence suggesting that the elevated level of HNE induced in the inflammatory response plays an essential role in acute and chronic destructive lung diseases^3^,^5^,^24^. In the present study, we demonstrate for the first time a novel anti-inflammatory action of sirtinol by inhibition of HNE activity. In addition, our results also showed that sirtinol treatment markedly alleviated LPS-induced lung injury in mice.

Many experimental and clinical studies have indicated that enhanced activity of HNE is related to the pathogenesis of acute and chronic diseases^4^,^25^,^26^,^27^,^28^. The vigorous exocytosis of elastase from azurophilic (primary) granules in activated neutrophils, so-called neutrophil degranulation, is a major cause of direct or indirect destruction of surrounding tissues^5^. Neutrophil granules contain several stored antimicrobial proteins, proteases and metalloproteinases^29^. HNE, a serine protease of the chymotrypsin family, is a pivotal contributor to tissue or organ damage in chronic inflammatory disease^25^,^26^,^30^. Hence, HNE has emerged as a therapeutic target in many inflammatory diseases. Sirtinol efficiently suppressed HNE activity in activated human neutrophils in response to various stimulators. These results support our hypothesis that sirtinol has the potential to be a useful anti-inflammatory drug.

In addition to HNE, respiratory burst also plays a critical role in mediating neutrophil functions. The production of superoxide, a precursor of other reactive oxygen species, plays an important role in oxidative tissue damage. Our study showed that sirtinol had no significant effect on superoxide generation in activated human neutrophils, and that it displayed no antioxidant ability in a cell-free system.

The level of HNE activity by activated human neutrophils can be reduced by modulating cellular signaling pathways, but also by directly inhibiting enzymatic activity. The signal transduction pathways in activated neutrophils that are responsible for HNE release are very complex and elusive. fMLF, a bacterial peptide, triggers neutrophils via binding to membrane-located G-protein coupled receptor (GPCR)^31^. When GPCR is activated, it can stimulate phospholipase C to increase the [Ca^2+^]_i_. Alternatively, the phosphatidylinositol 3-kinase (PI3K) pathway can cause neutrophil activation after GPCRs are triggered^32^,^33^. When PI3K is activated, Akt is recruited to the neutrophil membrane, and is phosphorylated and activated there to induce degranulation^34^. In addition, MAPKs and Src family kinase pathways all play a major role in neutrophil activation^31^,^35^,^36^. In contrast, the elevation of cAMP/PKA levels and dephosphorylation of proteins by PPs have negative regulatory effects on human neutrophil activation^15^,^16^,^17^,^18^,^20^. Surprisingly, our data showed that none of these signals is involved in the sirtinol-induced reduction in HNE activity in fMLF-activated cells. Based on these results, we hypothesize that sirtinol may act as an HNE inhibitor that does not affect other cell functions in activated human neutrophils. Our data also confirm that sirtinol inhibits, in a concentration-dependent manner, the activity of extracellular HNE obtained from the supernatant of cell cultures. The enzymatic activity assay using purified HNE also showed that sirtinol concentration-dependently inhibits HNE activity. To the best of our knowledge, this is the first study to demonstrate that sirtinol is an HNE inhibitor.

To validate these findings in vivo, we evaluated the therapeutic potential of sirtinol as a selective HNE inhibitor using the HNE-induced paw edema model in rodents. HNE can cause similar paw edema to other stimuli such as carrageenan and bradykinin^37^. The edematogenic effect in mouse paws is contributed to by HNE-induced increases in the permeability of the peripheral capillaries. The pathogenesis of ARDS is also characterized by abnormalities of vascular permeability in the lungs that lead to pulmonary edema, and is thought to be related to increased activity of HNE. Selective HNE inhibitors have been shown to suppress the increase in vascular permeability, neutrophil chemotaxis, and the formation of granulation tissues induced by stimuli^38^. In the present study, intraperitoneal injection with sirtinol effectively inhibited HNE-induced paw edema in mice in a dose-related manner. We also evaluated the beneficial effects of sirtinol on LPS-induced ALI in an animal model. Bacterial infection can result in sepsis, acute lung injury, and multiple organ failure, consequently leading to a high mortality rate. LPS can elicit strong immune responses in animals, and is recognized as a potent toxin that can initiate the pathogenesis of endotoxic shock or organ injury with a high mortality^39^. The severity of lung injury caused by LPS is related to the enhanced sequestration of neutrophils in the lungs^40^. Our study showed that sirtinol may reduce neutrophil recruitment and pulmonary edema in mice subjected to LPS-induced ALI. Activated neutrophils secrete large amounts of cytotoxins, and play a central role in the development of septic shock and organ failure^41^, and NE-deficient mice are resistant to LPS-induced lethal infections^42^. These studies identified that HNE could be an effector in endotoxic shock and organ failure. Sirtinol probably has the potential for use in clinical therapy as a novel adjunct in the treatment of pulmonary edema or ARDS related to overactivity of HNE.

**Figure 9:**
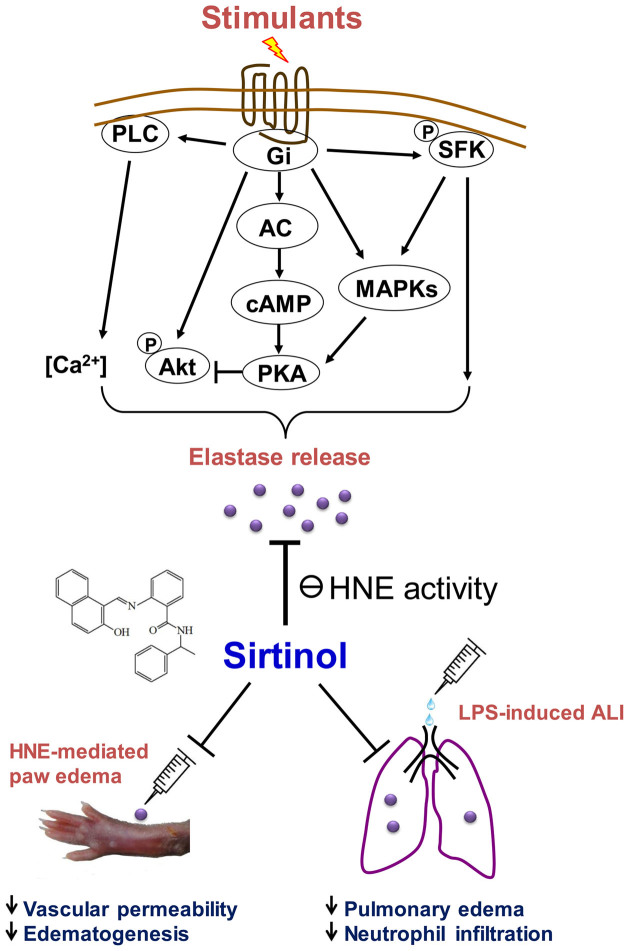
Schematic diagram illustrating the anti-inflammatory effects of sirtinol in vitro and in vivo. Sirtinol directly inhibits the enzymatic activity of HNE released from activated human neutrophils, and protects against HNE-induced paw edema and LPS-caused ALI in mice.

In conclusion, the present study is the first to identify sirtinol as an NE inhibitor, and to demonstrate its protective effects in HNE-induced paw edema and LPS-mediated ALI in mice (Fig. 9). ALI and ARDS remain the major causes of morbidity and mortality in critically ill patients. Our evidence suggests that sirtinol may in future be applied to the treatment of HNE-driven lung inflammation.

## Methods

### Preparation of human neutrophils

Blood samples were obtained from healthy volunteers aged 20–30 years old after written informed consent was obtained. The study protocol was investigated and approved by the Institutional Review Board at Chang Gung Memorial Hospital. The methods were carried out in accordance with the approved guidelines. Neutrophils were isolated by standardized procedures that included dextran sedimentation, gradient centrifugation using Ficoll-Hypaque, and hypotonic lysis of erythrocytes. Purified neutrophils were more than 98% viable as calculated by the Trypan blue exclusion technique. The cells were suspended in ice cold Ca^2+^-free Hank's balanced salts solution (HBSS).

### Determination of superoxide production

Reduction of ferricytochrome *c* was monitored as a measure of superoxide production from activated neutrophils. After addition of 0.5 mg/mL ferricytochrome *c* and 1 mM Ca^2+^ to neutrophils (6 × 10^5^ cells/mL), they were equilibrated at 37°C for 5 min and then preincubated with DMSO or sirtinol. Neutrophils were then stimulated with 30 nM fMLF, 0.3 μM MMK1, or 5 nM PMA. Cytochalasin B (CB) at 1 μg/mL was added 3 min before fMLF or MMK1 stimulation. We continuously monitored the change in absorbance at 550 nm with a spectrophotometer (U-3010; Hitachi, Tokyo, Japan). Results were calculated as previously described^43^.

### Determination of HNE activity

Methoxysuccinyl-Ala-Ala-Pro-Val-p-nitroanilide (100 μM) as the substrate for HNE was mixed with human neutrophils (6 × 10^5^ cells/mL) at 37°C for 5 min. Then, the human neutrophils were preincubated with DMSO or sirtinol, and were activated with 30 nM fMLF after pretreatment with 0.5 μg/mL CB for 3 min. In addition, we also stimulated neutrophils with MMK1 (0.3 μM) or platelet activating factor (PAF; 1 μM). The change in absorbance at 405 nm was assayed continuously with a spectrophotometer. Results are expressed as a percentage of HNE activity in the stimulant-activated drug-free control group.

To test whether sirtinol is able to directly inhibit HNE activity, an activity assay of HNE was modified for a cell-free system. Human neutrophils (6 × 10^5^ cells/mL) were activated by addition of fMLF (100 nM) in the presence of CB (2.5 μg/mL) for 15 min at 37°C. HNE was obtained from the supernatant of cells after they were centrifuged at 1000 g for 5 min at 4°C. Then, the supernatant was equilibrated at 37°C for 2 min and incubated with or without sirtinol for 5 min. After incubation, HNE substrate (100 μM) was added to the reaction solutions. The changes in absorbance were continuously monitored for 10 min at 405 nm to determine the HNE activity.

### Assay of enzymatic activity of HNE

Twenty-five microliters of the substrate solution (500 μM methoxysuccinyl-Ala-Ala-Pro-Val-p-nitroanilide in 20 mM Tris-HCl buffer, pH 7.4) was mixed with different concentrations of sirtinol solution. For the assay, 25 μL of enzyme solution (200 nM of HNE) was added to 50 μL of sirtinol solution, and the resultant mixture was shaken well for 5 min in the dark. Subsequently, the 25 μL of substrate solution at a final concentration of 125 μM was added, and the mixture was incubated in an automatic CO2 incubator at 30°C. The rate of enzymatic hydrolysis of the substrate was assayed by increased absorbance at 405 nm using an automatic microplate reader. The absorbance values were determined by calculating the area under the curve.

### Determination of intracellular calcium concentration ([Ca^2+^]_i_)

After being labeled with 2 μM Fluo-3/AM at 37°C for 35 min, human neutrophils (3 × 10^6^ cells/mL) were washed and resuspended in HBSS. The suspension was treated with DMSO or sirtinol for 5 min in a quartz chamber, and then stimulated by 30 nM fMLF at 37°C. [Ca^2+^]_i_ was analyzed by measuring the change in fluorescence with a spectrofluorimeter (Hitachi F-4500) with continuous stirring. The excitation wavelength was set at 488 nm and the emission wavelength at 520 nm. [Ca^2+^]_i_ was calculated as [Ca^2+^]_i_ (nM) = K_d_ × (F − F_min_)/(F_max_ − F), where F is the peak value of detected fluorescence intensity in the fMLF-induced spike, and K_d_ (dissociation constant) is known to be 400 nM. F_max_ and F_min_ were measured when 0.05% Triton X-100 and EGTA (20 mM), respectively, were added to the cells.

### Western blot of whole-cell lysates

Human neutrophils were incubated with DMSO or sirtinol (3–20 μM) at 37°C for 5 min, and then activated by 30 nM fMLF for another 30 s after pretreatment with 1.0 μg/mL CB for 3 min. Sample buffer (62.5 mM pH 6.8 Tris-HCl, 4% sodium dodecyl sulfate (SDS), 5% β-mercaptoethanol, 2.5 mM Na_3_VO_4_, 0.00125% bromophenol blue, 10 mM di-N-pentyl phthalate, and 8.75% glycerol) was injected to stop the reaction and the mixtures were heated at 100°C for 15 min. After being heated, the whole-cell lysates were centrifuged at 4°C for 20 min at 14,000 g, and the supernatant was used for immunoblotting assays. The target protein was identified by immunoblotting with the corresponding antibody overnight at 4°C, followed by incubation with horseradish peroxidase-conjugated, secondary anti-rabbit or anti-mouse antibody (Cell Signaling Technology, Beverly, MA). The labeled protein was measured using an enhanced chemiluminescence system (Amersham Biosciences). Bands on the blots were analyzed using a densitometer (UVP, Upland, CA), and the quantitative ratios for all samples were normalized to the corresponding total protein or to β-actin.

### HNE-induced paw edema

All animal experiments were performed in accordance with the guidelines of the Animal Welfare Act and The Guide for Care and Use of Laboratory Animals from the National Institutes of Health. Experimental protocols were approved by the Institutional Animal Care and Use Committee of Chang Gung Memorial Hospital. All C57BL/6 mice were purchased from CD® IGS, BioLasco, Ilan, Taiwan. Animals were kept in an air-conditioned room with light and dark cycles alternating every 12 h, and adapted to the environment for at least 1 week before the experiment.

30 male mice (20–25 g, 7–8 weeks old) were used in this model. The procedure for elastase-induced paw edema was performed as described previously^44^. Briefly, mice were randomly divided into five groups; then mice were intraperitoneally injected with 50 μL DMSO (vehicle group) or sirtinol (2.5 or 5.0 mg/kg body weight). After 1 h, paw inflammation was induced by injecting 25 μL of 200 μg/mL HNE (Enzo, USA) or an equal volume of 0.9% saline (sham group) into the intraplantar space of the right hind paw under anesthesia with Zoletil 50 (30 mg/kg) and xylazine (6 mg/kg). Before injection of HNE, the thickness of the right hind paw was measured using a digital caliper (Type 1108-150, INSIZE, Austria), as a baseline reference^44^. The thickness of the paw was then measured at the indicated times after HNE or saline injection. Five consecutive measurements were performed, and the maximum and the minimum were excluded. Edema was calculated as the average difference in paw thickness (mm) compared with the data for the sham group and the corresponding baseline reference.

### LPS-induced ALI

Twenty-four C57BL/6 male mice were randomly allocated to four groups. All mice were injected with sirtinol (5 mg/kg body weight, 50 μL intraperitoneally) or the same volume of DMSO. The four groups were (I) sham-operated mice injected with vehicle, (II) sham-operated mice injected with sirtinol, (III) ALI animals injected with vehicle, and (IV) ALI animals injected with sirtinol. One hour after administration, the mouse trachea was exposed by blunt dissection and a tracheostomy was performed under anesthesia. Acute lung injury was induced by gently instilling 50 μL of 3.2 mg/mL LPS from Escherichia coli serotype 055:B7 or 0.9% saline via the tracheostomy. At 6 h after instilling LPS or normal saline into the trachea, the animal lungs were removed after opening the chest. The left upper lobe of the lungs was analyzed for tissue water content, and two sections of right lower lobe were fixed in 10% formalin for histological examination. The right upper lobe was assayed for MPO activity. The extent of pulmonary edema was calculated as a wet- over dry-weight ratio compared with sham-treated animals.

### Assessment of lung MPO activity

MPO activity was assayed as an index of neutrophil infiltration in mouse lung that was subjected to LPS-induced ALI. Frozen lung tissues were thawed and immersed in 50 mM phosphate buffer containing 0.5% hexadecyltrimethylammonium bromide. Lung tissue samples were sonicated on ice using a homogenizer, and the homogenate was then centrifuged at 2,000 g for 15 min at 4°C. The MPO activity of homogenates was determined by adding them to phosphate buffer (pH 6.0) containing 0.167 mg/mL o-dianisidine hydrochloride and 0.0005% hydrogen peroxide (Sigma). The light absorbance was monitored at 460 nm by a spectrometer for 5 min according to the kit instructions. MPO activity was calculated by comparing results with the standardized concentration curve derived from commercial human MPO (Sigma). Final values were all normalized to the corresponding protein concentration. A Bio-Rad assay kit (Hercules, CA) was used to detect the protein content of every sample. Lung MPO activity was finally expressed in units per gram of tissue.

### Statistical analysis

Data are presented as means ± S.E.M. Statistical analysis was performed with SigmaPlot (Systat Software, San Jose, CA) using Student's *t*-test, and one- or two-way analysis of variance followed by Bonferroni's multiple-comparison test. A significant difference was presumed when *p* < 0.05.

## Author Contributions

Y.F.T. and H.P.Y. performed most in vitro assays and animal studies. W.Y.C., F.C.L. and Z.C.H. carried out calcium concentration analysis, cytotoxicity assay, and enzymatic activity assay. T.L.H. and Y.F.T. were responsible for the overall design, analyzed data, and wrote the article.
